# Gallic Acid Prevents the Oxidative and Endoplasmic Reticulum Stresses in the Hippocampus of Adult-Onset Hypothyroid Rats

**DOI:** 10.3389/fphar.2021.671614

**Published:** 2021-07-06

**Authors:** Vanessa Blas-Valdivia, Margarita Franco-Colín, Placido Rojas-Franco, Alberto Chao-Vazquez, Edgar Cano-Europa

**Affiliations:** ^1^ Lab. Neurobiología, Departamento de Fisiología “Dr. Mauricio Russek Berman”, Escuela Nacional de Ciencias Biológicas, Instituto Politécnico Nacional, Ciudad de México, Mexico; ^2^ Lab. de Metabolismo I, Departamento de Fisiología “Dr. Mauricio Russek Berman”, Escuela Nacional de Ciencias Biológicas, Instituto Politécnico Nacional, Ciudad de México, Mexico

**Keywords:** gallic acid, hypothyroidism, oxidative stress, endoplasmic reticulum stress, hippocampus

## Abstract

Thyroid hormone is essential for hippocampal redox environment and neuronal viability in adulthood, where its deficiency causes hypothyroidism related to oxidative and endoplasmic reticulum stresses in the hippocampus, resulting in neuronal death. One option of treatment is antioxidants; however, they must be transported across the blood-brain barrier. Gallic acid is a polyphenol that meets these criteria. Thus, this study aimed to prove that the neuroprotective mechanism of GA is associated with the prevention of oxidative and endoplasmic reticulum stresses in the hippocampus of adult-onset hypothyroid rats. Male Wistar rats were divided into euthyroid (n = 20) and hypothyroid groups (n = 20). Thyroidectomy with parathyroid gland reimplementation caused hypothyroidism. Each group was subdivided into two: vehicle and 50 mg/kg/d of gallic acid. 3 weeks after thyroidectomy, six animals of each group were euthanized, and the hippocampus was dissected to evaluate oxidative and endoplasmic reticulum stress markers. The rest of the animals were euthanized after 4 weeks of treatment for histological analysis of the hippocampus. The results showed that hypothyroidism increased lipid peroxidation, reactive oxygen species, and nitrites; it also increased endoplasmic reticulum stress by activating the inositol-requiring enzyme-1α (IRE1α) pathway, the protein kinase RNA-like endoplasmic reticulum kinase (PERK) and activated transcription factor 6α (ATF6α) pathways associated with a proapoptotic state that culminates in hippocampal neuronal damage. Meanwhile, the hypothyroid rat treated with gallic acid reduced oxidative stress and increased endoplasmic reticulum-associated degradation (ERAD) through IRE1α and ATF6. Also, the gallic acid treatment prevented the Bax/BCl2 ratio from increasing and the overexpression of p53 and caspase 12. This treatment in hypothyroid animals was associated with the neuronal protection observed in the hippocampus. In conclusion, gallic acid prevents hypothyroidism-induced hippocampal damage associated with oxidative and endoplasmic reticulum stresses.

## Introduction

Thyroid hormones are crucial for brain development in the perinatal age. Meanwhile, in adulthood, they participate in the steady-stable state of brain function (Carageorgiou et al., 2005; Cano-Europa et al., 2008; Alva-Sánchez et al., 2009). Hypothyroidism is a common pathological condition of thyroid hormone deficiency, and it is not enough to satisfy the metabolic demand of all tissue that depends on the hormone action. In the brain, the clinical manifestation of hypothyroidism includes neurologic symptoms like memory impairment, anxiety, dementia, and depression, among others (Samuels, 2014). One of the brain regions involved in those symptoms is the hippocampus, which is sensitive to thyroid hormone deprivation (Koromilas et al., 2010). In adult animals, this region is susceptible to hypothyroidism-modifies cholinergic, noradrenergic, opioidergic, and glutamatergic neurotransmission (Ortiz-Butron et al., 2003; Carageorgiou et al., 2005; Ghosh and Das, 2007). Several studies have shown that hypothyroidism-induced hippocampal damage is associated with the changes in the glutamatergic neurotransmission producing the reduction in the glutamate release (Sánchez-Huerta et al., 2012), and the overexpression of kainate receptors (Giné et al., 2010) and the subunits NR2A and NR2B of N-methyl-D-aspartate receptor (Lee et al., 2003). All these physiological events promote hippocampal neuronal damage through glutamate excitotoxicity (Alva-Sánchez et al., 2009) with the consequent increase of oxidative (Cano-Europa et al., 2008) and endoplasmic reticulum stresses (Torres-Manzo et al., 2018). Recently, it has been demonstrated that hypothyroidism promotes neuroinflammation and dysregulation of glia-neuron communication. This pathophysiological situation induces hippocampal apoptosis through autophagy (Mishra et al., 2021).

The association between neuroinflammation, oxidative and endoplasmic reticulum stresses promotes neuronal damage. Under normal physiological conditions, reactive oxygen species (ROS) and reactive nitrogen species (RNS) activate signaling pathways to maintain a steady-stable state in the cell. Oxidative stress occurs when the generation of oxidants exceeds their capability to neutralize them by the antioxidant systems. Cellular impairment occurs through lipid peroxidation, protein oxidation, DNA damage, and mitochondrial and endoplasmic reticulum (ER) function inhibition. Therefore, it is unsurprizing that pathological alterations culminate in endoplasmic reticulum stress (ERS), manifested as disturbed protein folding and accumulation of unfolded proteins in the ER lumen (Xu et al., 2005). Thus, the ER triggers the unfolded protein response (UPR), which tries to restore protein homeostasis through the protein kinase RNA-like endoplasmic reticulum kinase (PERK) pathway. PERK promotes the phosphorylation of eukaryotic initiation factor 2α (eIF2α), reducing the global protein synthesis except for selective mRNAs as activating transcription factor 4 (ATF4). ATF4 regulates genes involved in the redox environment maintenance and antioxidant enzymatic system, which are crucial for the cell adaptation to stress conditions (Chakravarthi et al., 2006). Another pathway to prevent ERS is activating the transcription factor 6 (ATF6) pathway that up-regulates gene expression responsible for mediating protein folding and ER-associated protein degradation (ERAD) mechanism. Thus, ERS can activate autophagy as a mechanism to prevent the build-up of toxic protein aggregates, removed organelles as ER, and alleviate cell stress. However, during long-term ERS, ATF4 stimulates CCAAT-enhancer-binding protein homologous protein (CHOP), also named growth arrest- and DNA damage-inducible gene 153 (GADD153), which paradoxically is the responsible effector for the initiation of the apoptotic process (Nishitoh, 2012). When ERS is not alleviated, the cell decides to die through the overexpression of GADD153 and the activation of the inositol regulating enzyme 1α (IRE1α) pathway, increasing Bax and caspase 12 with a reduction in the Bcl2 expression (Sprenkle et al., 2017). Finally, ERS promotes neuronal death through autophagy activated by PERK and IRE1α (Kouroku et al., 2007; Liu C. et al., 2020).

Natural products from plants have been efficient molecules with neuroprotective activities to treat neurological diseases. One molecule with high potential is gallic acid (3,4,5-trihydroxy benzoic acid), a low molecular weight triphenolic compound ubiquitously found in almost all parts of the plant, including seed, root, fruit, leaf, wood, and bark, which is well absorbed and transported across the blood-brain barrier compared with other polyphenols (Badhani et al., 2015). Gallic acid (GA) possesses antioxidant, anti-inflammatory, and neuroprotective activities (Samad et al., 2019; Mirshekari Jahangiri et al., 2020). This molecule can be used to treat different neurological diseases that cause neuronal damage. However, some research groups proposed that GA prevents oxidative and neuronal damage because it reduces glial activation, neuroinflammation, and protein aggregation (Liu Y.-L. et al., 2020). Thus, this study aimed to prove that the neuroprotective mechanism of GA is associated with the prevention of oxidative and endoplasmic reticulum stresses in the hippocampus of adult-onset hypothyroid rats.

## Methods

### Animals

Forty male Wistar rats (200–250 g) of our care facilities were kept in acrylic cages (80 cm × 30 cm × 20 cm) in a cooled-regulated room (20 ± 1°C) with water and food *ad libitum*, light cycles of 12/12 h and relative humidity of 40–60%.

Animals were divided into hypothyroid (*n* = 20) and euthyroid (*n* = 20). Hypothyroidism was induced by thyroidectomy with parathyroid reimplantation, as previously described (Cano-Europa et al., 2010; Torres-Manzo et al., 2018). After surgery, four groups were formed, each one with ten animals: 1) euthyroid + vehicle (0.9% saline solution with 0.05% acetic acid at 40°C), 2) euthyroid +50 mg/kg/d gallic acid, 3) hypothyroid + vehicle and 4) hypothyroidism + gallic acid. Each day the GA (Sigma-Aldrich G7384) was prepared, and the treatment was administrated by oral gavage at 9:00 h. 3 weeks after treatment, six animals of each group were euthanized by decapitation, the brain was removed, and the hippocampus dissected were frozen at −80°C until used. A serum sample was used to determine thyroid hormones (T_3_ and T_4_) (ELISA kit form Diagmex, México). 4 weeks after surgery, four animals of each group were transcardially perfused under pentobarbital anesthesia (35 mg/kg ip) with 0.9% saline and 10% formaldehyde. The brains of the rats were removed and were embedded in paraffin. Coronal cuts of 7 μm were obtained from −2.3 to −3.8 mm from Bregma (Paxinos and Watson, 1999). Each brain section was stained with toluidine blue. We evaluated the number of normal and abnormal neurons in the hippocampal CA3 within a 10,000 μm^2^ section as previously described (Blas-Valdivia et al., 2007; Lopez-Galindo et al., 2008).

### Oxidative and Endoplasmic Reticulum Stresses Evaluation

The dissected hippocampus was homogenized in 500 μl 10 mM phosphate buffer pH 7.4. It was used to perform all determinations of lipid peroxidation, nitrites, and reactive oxygen species (ROS) quantification as previously described (Torres-Manzo et al., 2018).

Western blot samples were prepared as previously described (Torres-Manzo et al., 2018). Briefly, 50 μg of protein charged in 15% polyacrylamide gels and separated by electrophoresis (120 mV for 120 min). Then electrotransferred to PVDF membranes using a Trans-Blot Turbo Transfer System (Biorad) for 9 min (25 V, 2.5 A). After that, membranes blocked and incubated at −4°C overnight with the primary antibodies. The primary antibody obtained from Santa Cruz Biotechnology (Dallas, Texas) were dilute 1:100. The primary antibodies from this manufacturer were: caspase 12 (sc-21747), ATF-6α (sc-166659), XBP1(sc-8015), GADD153 (sc-56107), eIF2α (sc-133132), *p*-eIF2α (Ser 52) (sc-12412), IRE1α (sc-390960), ATF-4 (sc-sc-390063), Bax (sc-20067), Bcl2 (sc-7382), PERK (sc-377400). Also, we used two primary antibodies from Biorbyt (Cambridge, England), GADD34 (orb183811) and p53 (Orb14498) diluted 1:500. After incubation, membranes washed three times and then incubated in 1:2000 diluted secondary antibody from Thermo Fisher Scientific (HPR-conjugated goat anti-rabbit (31,466), HPR-conjugated rabbit anti-goat (A16136); HPR-conjugated goat rabbit anti-rat (A18915) or Santa Cruz (HPR-conjugated goat mouse, sc-2302) for 1 h at room temperature, under constant stirring. Then, membranes washed three times and protein bands revealed in photographic plates (JUAMA, México) by chemiluminescence, using Luminata TM Forte^®^ (Millipore). Protein β-actin expression used as a constitutive protein (Santa Cruz Biotechnology; sc-47778, dilution: 1:1500). Optical density from all bands obtained was analyzed by the ImageJ program (NIH, Bethesda, Maryland, United States; version 1.51p) according to program specifications, as previously we reported (Torres-Manzo et al., 2018).

Finally, caspase nine activity was assessed using a commercial colorimetric assay kit from Chemicon-Millipore (APT139). 5 μl of homogenate was added to a 96-well polystyrene plate mixed with 85 μl of caspase three substrate and 10 μl of LEHD-para-nitroanilide. The mix was incubated at 37°C, and absorbance at 405 nm was monitored for 1 h. Caspase activity is expressed as μmol of para-nitroanilide (p-NA) released/mg protein/h.

### Statistical Analysis

All data are presented as mean ± the standard error of the mean. Data analyzed by two-way analysis of variance (Two-way ANOVA) and the Tukey *post hoc* test. The factors were the treatment with GA and the thyroid state. Values that presented a *p* < 0.05 were considered statistically different.

## Results

**FIGURE 1:**
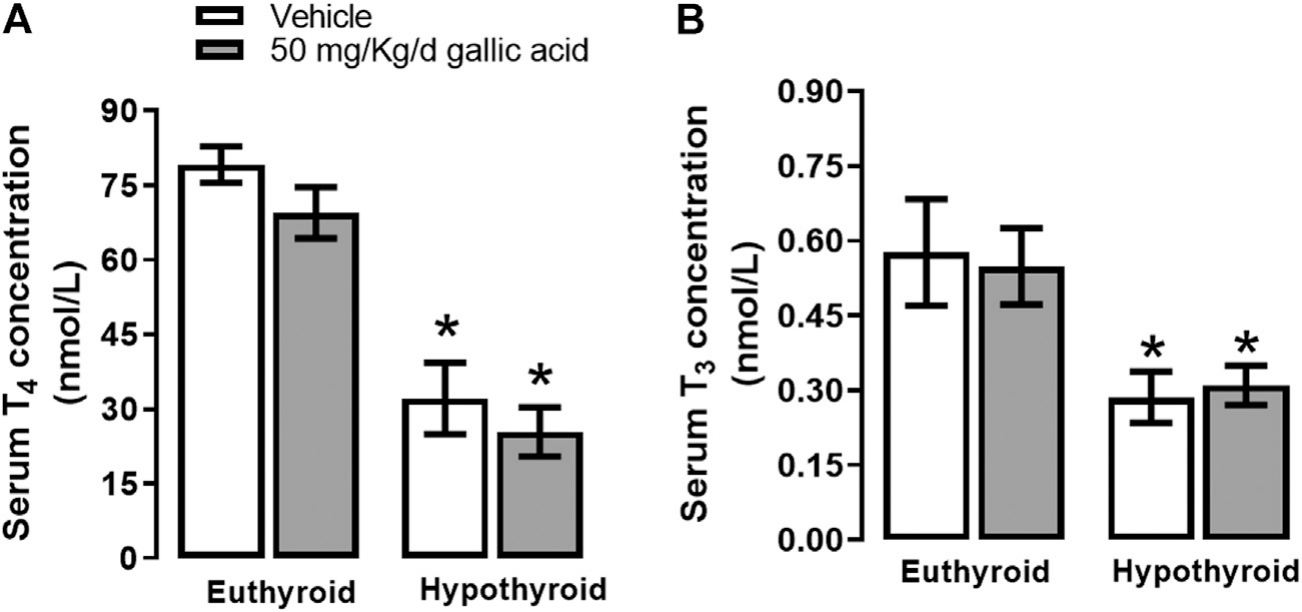
Effect of gallic acid on thyroid hormone concentration. Values represent the mean ± SEM. (*) *p* < 0.05 vs. Euthyroid group same treatment group.

Hypothyroidism induced by thyroidectomy with parathyroid reimplant reduced the T_3_ (panel A) and T_4_ (panel B) concentrations to euthyroid groups. Also, the gallic acid did not affect the thyroid hormone concentration in any group, as shown in Figure 1.

**FIGURE 2:**
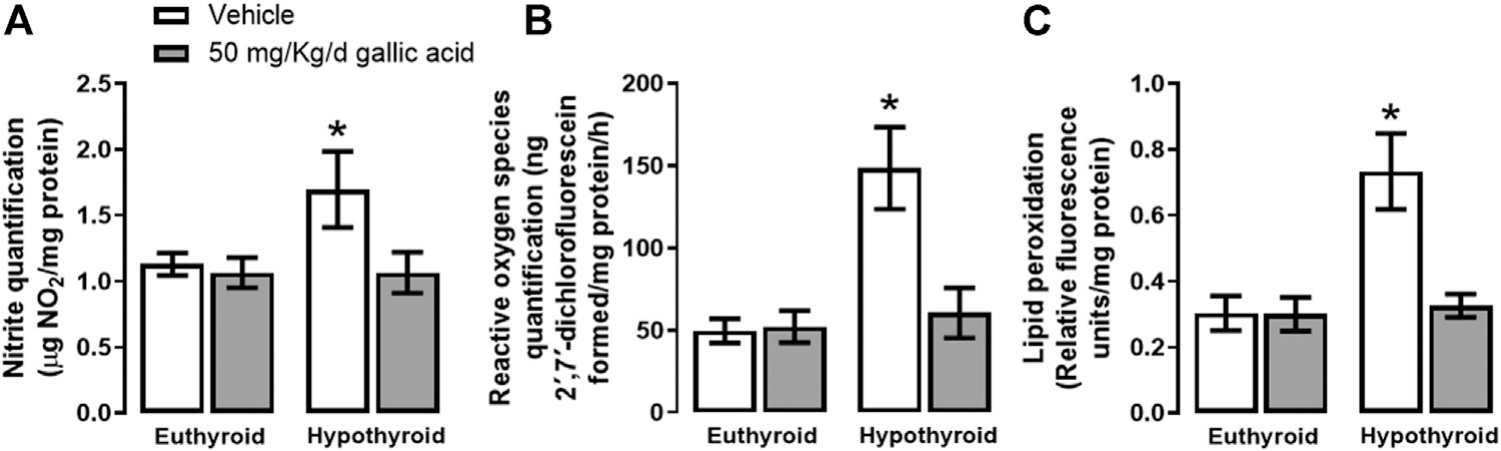
Effect of gallic acid on hypothyroidism-induced oxidative stress in the hippocampus of adult rats. The oxidative stress markers evaluated were nitrite and ROS quantifications **(panels A and B)** and lipid peroxidation **(panel C)**. Values represent the mean ± SEM. (*) *p* < 0.05 vs. Euthyroid + vehicle group.

Figure 2 shows that hypothyroidism causes oxidative and nitrosative stresses in the hippocampus of adult onset-hypothyroid rats because it increased nitrites (panel A), ROS (panel B), and lipid peroxidation (panel C) after three thyroidectomy weeks. Meanwhile, the gallic acid treatment prevented the increase of all these markers.

**FIGURE 3:**
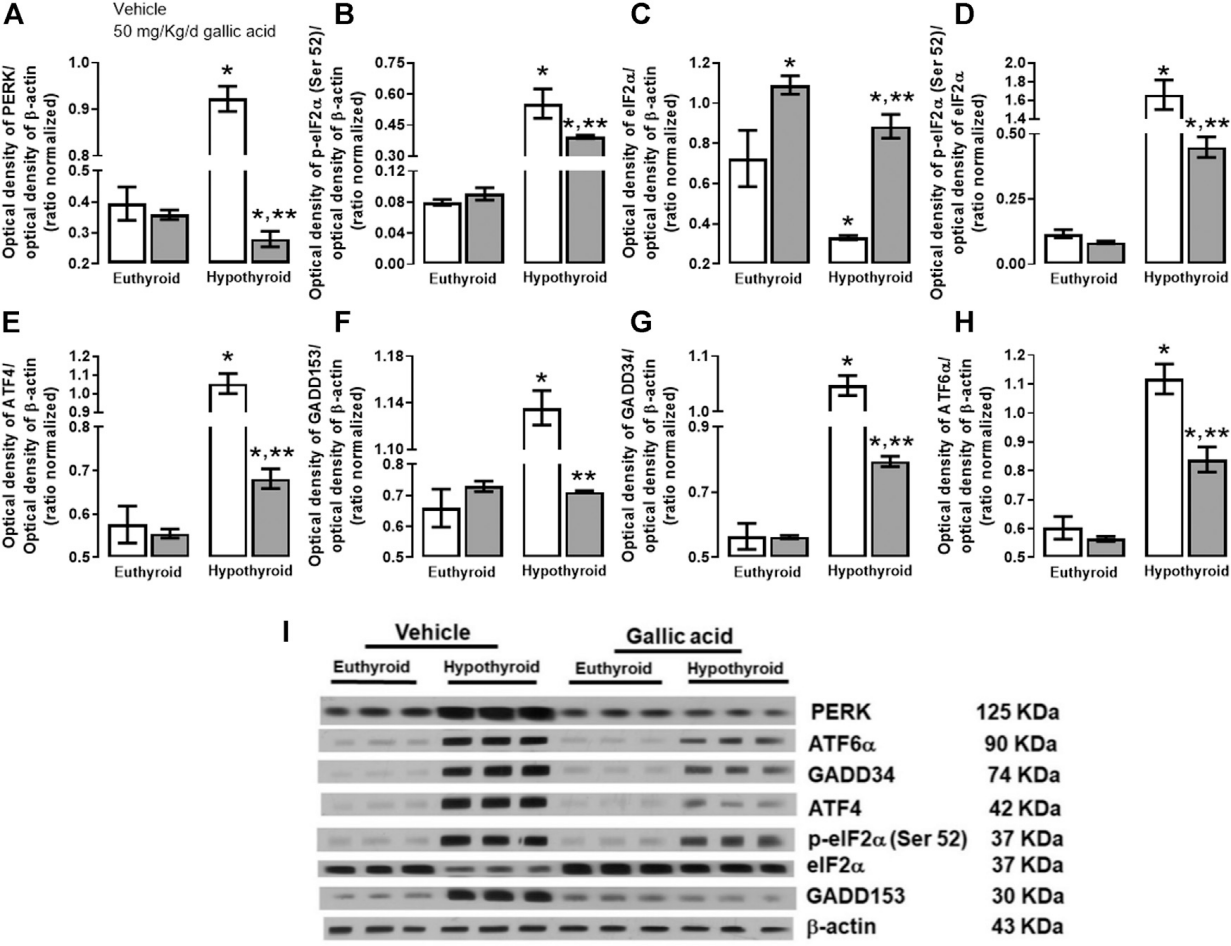
Effect of gallic acid on hypothyroidism-induced endoplasmic reticulum stress through PERK-eIF2α-ATF4-GADD153 pathway in the hippocampus of adult rats. The protein expresión evaluated were PERK **(A)**, *p*-eIF2α (Ser 52) **(B)**, eIF2α **(C)**, ATF4 **(E)**, GADD153 **(F)**, GADD34 **(G)**, ATF6α **(H)** and the *p*-eIF2α (Ser 52)/eIF2α ratio **(D)**. **(I)** shows the blot of the protein expression. Values represent the mean ± SEM. (*) *p* < 0.05 vs. Euthyroid group same treatment group. (**) *p* < 0.05 vs. hypothyroid + vehicle group.

**FIGURE 4:**
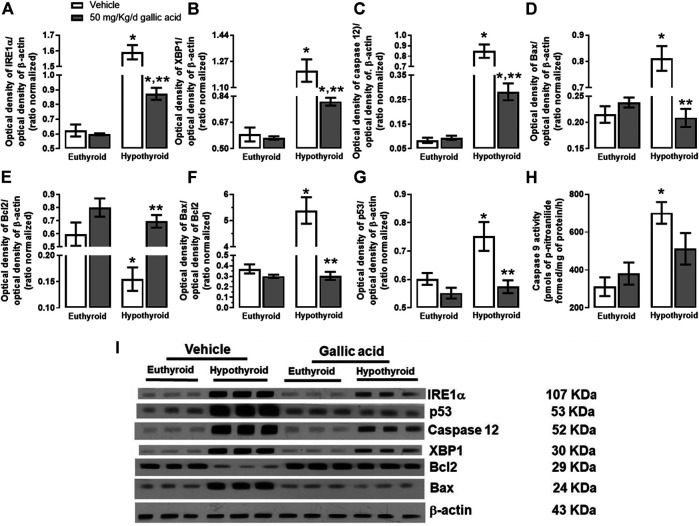
Effect of gallic acid on hypothyroidism-induced activation of the endoplasmic reticulum stress pathway related to neuronal damage in adult rats’ hippocampus. The protein expression evaluated were IRE1α **(A)**, XBP1 **(B)**, caspase 12 **(C)**, Bax **(D)**, BCl2 **(E)**, p53 **(G)**, and the Bax/Bcl2 ratio **(F)**. **(H)** shows the activity of caspase 9, and panel I, the blot of the protein expression. Values represent the mean ± SEM. (*) *p* < 0.05 vs. Euthyroid + vehicle group. (**) *p* < 0.05 vs. hypothyroid + vehicle group.

The evaluation of the canonical pathway of endoplasmic reticulum stress PERK-eIF2α-ATF4-GADD153 is shown in Figure 3. Hypothyroidism increased in the hippocampus the expression of PERK (134%, panel A), p-eIF2α−(Ser 52)/eIF2α (592%, panel B), the relation p-eIF2α−(Ser 52)/eIF2α (1331%, panel D). ATF4 (204%, panel E), GADD153 (72%, panel F), GADD34 (72%, panel G) and ATF6α (85%, panel H). Moreover, hypothyroidism reduced the expression of eIF2α (54%, panel C) compared with the euthyroid condition. However, hypothyroid animals which received treatment with gallic acid normalized the expression of f PERK and GADD153, as well as, it partially prevented the alteration of peIF2α−(Ser52), eIF2α, GADD34, ATF4, and ATF6α expression. The effect of gallic acid on IRE1 α pathway and the neuronal death markers caused by adult-onset hypothyroidism in the hippocampus is depicted in Figure 4. Hypothyroidism induced an overexpression of IRE1α (155%, panel A), XPB1 (104%, panel B), caspase 12 (909%, panel C), Bax (277%, panel D), the ratio Bax/Bcl2 (1354%, panel G), p53 (24%, panel G) and overactivated caspase nine by about 125% (panel H). Also, hypothyroidism down-expressed Bcl2 by about 74% (panel A). On the other hand, the expression of Bcl2, Bax, the ratio Bax/Bcl2, p53, and the activity of caspase nine were normalized in the hippocampus of hypothyroid rats treated with gallic acid. This treatment also prevented hypothyroidism-induced overexpression of IRE1α, XBP1, and caspase 12 (40, 41, and 200% respectively compared with the euthyroid group).

**FIGURE 5:**
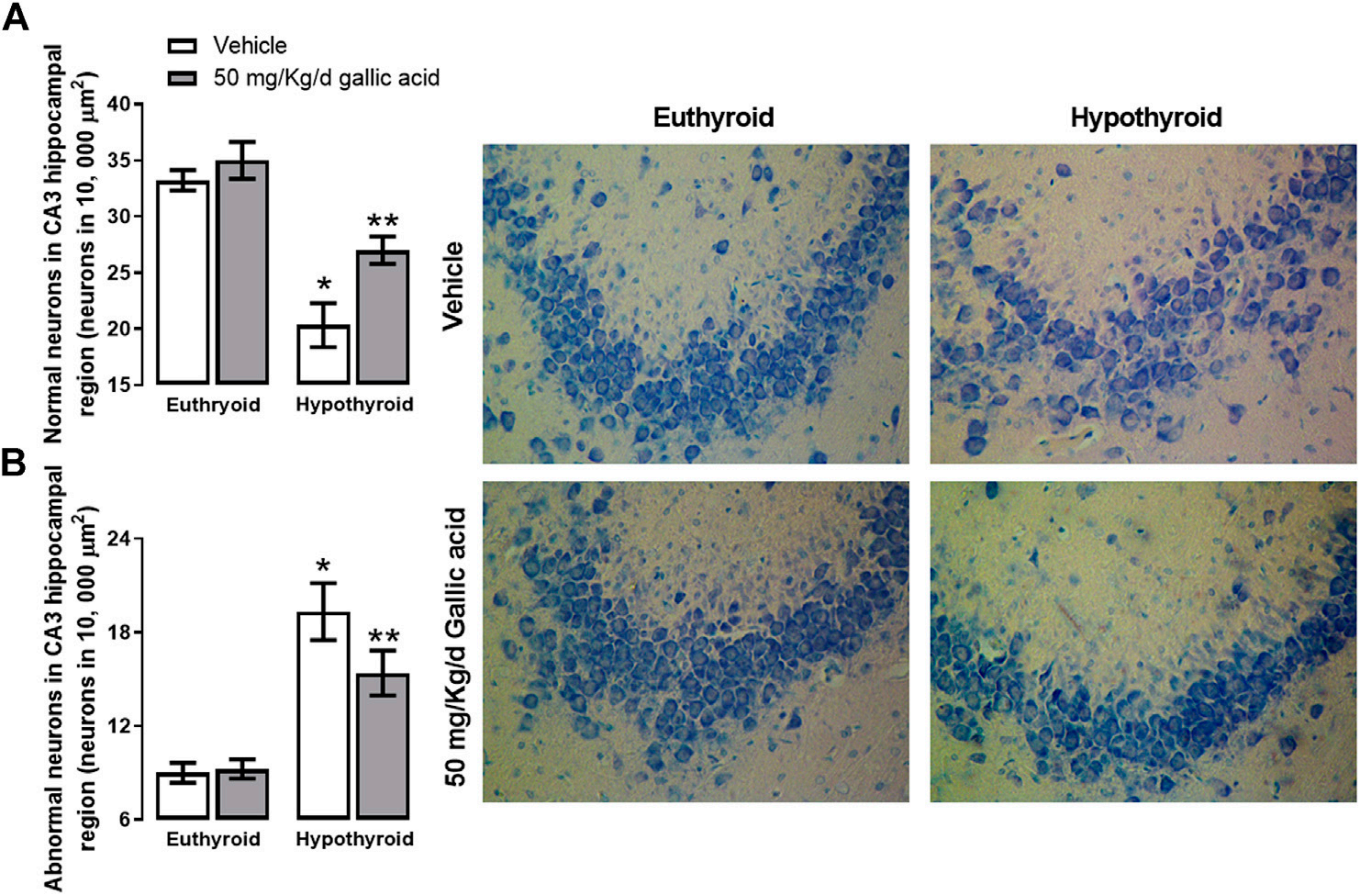
Effect of gallic acid on hypothyroidism-induced morphological alteration in CA3 hippocampal region of adult rats. It shows normal **(A)** and abnormal neurons **(B)**. In the figure’s right, there are representative photomicrographs of the treated groups stained with toluidine blue. Values represent the mean ± SEM. (*) *p* < 0.05 vs. Euthyroid + vehicle group. (**) *p* < 0.05 vs. hypothyroid + vehicle group.

These findings were also corroborated by histological examination of the hippocampal section shown in Figure 5. Hypothyroidism reduced the normal morphological neurons by about 37%, and it increased the abnormal morphological neurons by about 111% to the euthyroid group after four weeks post-thyroidectomy. However, in the hypothyroid group treated with gallic acid, the normal morphological neurons’ reduction was about 22%, and the increase of abnormal morphological neurons was about 66%.

## Discussion

The hippocampus is a brain region susceptible to neuronal damage due to onset-adult hypothyroidism. The mechanism involved the change in neurotransmission, particularly the glutamatergic pathways (Alva-Sánchez et al., 2009; Giné et al., 2010; Sánchez-Huerta et al., 2012) that causes oxidative and endoplasmic reticulum stresses (Torres-Manzo et al., 2018), neuroinflammation and autophagy (Mishra et al., 2021). Nevertheless, it is crucial to develop a treatment to avoid the neuronal damage due to this disease. There are reports on natural products with potential applications on oxidative stress-caused damage in different diseases. For example, polyphenols are secondary metabolites from plants with antioxidants, anti-inflammatory, and neuroprotective activities. One of the most studied polyphenols is GA which exerts all the effects mentioned above. GA provides efficient neuroprotection against oxidative damage (Badhani et al., 2015) because it reduces glial activation and protein aggregates (Liu Y.-L. et al., 2020). However, it still unknown if GA prevents endoplasmic reticulum stress (ERS). Thus, this study aimed to prove that the neuroprotective mechanism of GA is associated with the prevention of oxidative and endoplasmic reticulum stresses in the hippocampus of adult-onset hypothyroid rats.

First, we observed that hypothyroidism-induced endoplasmic reticulum stress in the third week after thyroidectomy because IRE1α, PERK, and ATF6α pathways are overactivated with a persistent proapoptotic state. We proposed that hypothyroidism causes chronic endoplasmic reticulum stress that culminates in neuronal damage in the CA3 hippocampal region. In this context, in the hippocampus of hypothyroidism rats, the ER-associated degradation (ERAD) is activated because ATFα was overexpressed. This transmembrane protein translocates into the nucleus to promote the expression of GADD34, GADD153, and XBP1. Further, hypothyroidism augmented the expression of ATF4 and GADD153, which activates gene encoding proteins to increase the synthesis of stressed cells leading to oxidative and nitrergic stresses into the cell, as we observed in previous results (Sprenkle et al., 2017; Torres-Manzo et al., 2018). Also, UPR was activated in the hippocampus of hypothyroid animals because the relation p-eIF2α−(Ser 52)/eIF2α was elevated to maintain proteostasis. In this way, UPR failed to avoid the protein synthesis reduction, although overexpression of GADD34 did not avoid dephosphorylation of p-eIF2α−(Ser 52). Recently, it was reported that hypothyroidism causes neuroinflammation (Nam et al., 2018) that can be associated with stimulating the IRE1α-TRAF-JNK pathway associated with caspase 12 and Bax-dependent apoptosis (Putcha et al., 2003; Sprenkle et al., 2017) where our result support this finding.

Moreover, hypothyroidism increased the PERK pathway promoting a decrease in the protein levels of IkB, which in turn favors the migration of NFkB into the nucleus to up-regulate the transcription of inflammatory genes (Deng et al., 2004). Activation of apoptotic mitogen-activated protein kinase (MAPK) p38-p53 pathway in astroglia increased IL-1β, IL6, and TNFα with a higher expression of COX2 (Nam et al., 2018). Neuroinflammation caused by hypothyroidism is associated with hippocampal autophagy (Mishra et al., 2021). Our results support this hypothesis because PERK/eIF2α activation promotes autophagosome elongation and maturation (Zheng et al., 2019).

This study is the first reported to correlate ERS and GA. Our results showed that GA *per se* only reduced Bcl2 expression in the hippocampus without affecting the hippocampal neuron. However, the hypothyroid animals treated with GA prevent neuronal damage in the CA3 region reducing oxidative, nitrergic, and endoplasmic reticulum stresses.

GA is a planar molecule consisting of an aromatic ring with three phenolic hydroxyl groups that scavenge ROS and RNS and avoid reducing first-line antioxidant enzymes’ activity to prevent oxidative stress (Badhani et al., 2015). GA scavenges reactive species and free radicals, avoiding neuronal death against different insults (Mirshekari Jahangiri et al., 2020; Schimites et al., 2020). Another beneficial effect of GA on neuronal excitotoxicity is reducing the glutamate release and intracellular calcium currents that avoid subcellular stress in the mitochondria and the endoplasmic reticulum (Ban et al., 2008). All these mechanisms can contribute that GA partially prevented neuronal death caused by hypothyroidism. We proposed that another neuroprotection mechanism of GA involves the prevention of ERS, where the results demonstrated that GA normalizes the expression levels of Bax, BCl2, and p53 in the hippocampus of hypothyroid rats. These results are in according to a previous report in the model of neurodegeneration caused by diabetes (Abdel-Moneim et al., 2017). Also, the GA association with the reduction of ERS is supported because misfolded proteins bind GRP78, promoting ROS increase and disulfide bond formation in the ER during the transfer of electrons from thiol groups in folding substrates (Sevier et al., 2007). An antioxidant like GA prevents this process by its scavenging action (Malhotra et al., 2008; Badhani et al., 2015). We propose that GA treatment participates in maintaining the proteostasis because IRE1α and ATF6 enhanced ERAD. After all, it attenuates the global synthesis of mRNA through the p-eIF2α−(Ser 52), which induces the expression of AT4. The activation of this response in hypothyroid animals avoids the disturbance in the redox environment due to the transcription of several prosurvival genes that encode chaperones and antioxidant enzymes (Sprenkle et al., 2017). GADD34 in the hypothyroid group treated with GA facilitated UPR response and activated the adaptation response through restoring proteostasis. This situation allows hippocampal neurons to restore the oxidative and protein stresses to avoid neuronal damage (Chakravarthi et al., 2006; Hetz et al., 2020).

Also, we proposed that GA promotes the eIF2α phosphorylation independently of PERK activation as it has been demonstrated that other polyphenols which activate GCN2 (General control nonderepressible 2) or PKR (Protein kinase RNA-activated) (Zhang et al., 2016; Nakagawa and Ohta, 2019). The reduction of PERK activation due to GA treatment prevents hypothyroidism which in turn causes autophagy.

Moreover, GA prevented IRE1α-caused hippocampal neuronal damage due to hypothyroidism; although IRE1α was over-expressed, it enhanced the ERAD response. In this group, GA prevented hypothyroidism-increased caspase 12, GADD153, ATF4, p53, Bax, and the activity of caspase 9. GA prevented the activation of the canonical pathway of cell death through Bcl2 family members. This idea is supported by the fact that GA prevents kainic acid-induced excitotoxic neuronal damage by reducing ROS and intracytoplasmic calcium current through JNK/p38/MAPK/p53 signaling pathway (Huang et al., 2012). The reduction in JNK/p38 pathway is associated with the anti-inflammatory activity of GA. Also, previous reports demonstrated that gallic acid prevents the hippocampal inflammation associated with neuronal damage (Diaz et al., 2020) and glial activation in culture cell that promotes protein aggregation and necroptosis (Siddiqui et al., 2019; Liu Y.-L. et al., 2020).

The main study limitation probably relies on the lack of specific effects of GA on glial cells (e.g. astrocytes, microglia). Thus, we envision to adress this specific topic in future studies as neuroinflammation is also a key-driver on hypothyroidism-induced hippocampal damage.

Finally, this study demonstrated that GA treatment prevents the oxidative and endoplasmic stresses associated with neuronal damage in the hippocampus of adult-onset hypothyroid rats.

## Data Availability

The raw data supporting the conclusion of this article will be made available by the authors, without undue reservation.
