# Supplementary material for: Gallic Acid Prevents the Oxidative and Endoplasmic Reticulum Stresses in the Hippocampus of Adult-Onset Hypothyroid Rats
Source: Front Pharmacol. 2021 Jul 6;12:671614. doi: 10.3389/fphar.2021.671614 (PMC8290492; doi:10.3389/fphar.2021.671614)
Supplement: Supplementary file 1 [file DataSheet1.ZIP › wester bloting.pptx]

## Slide 1
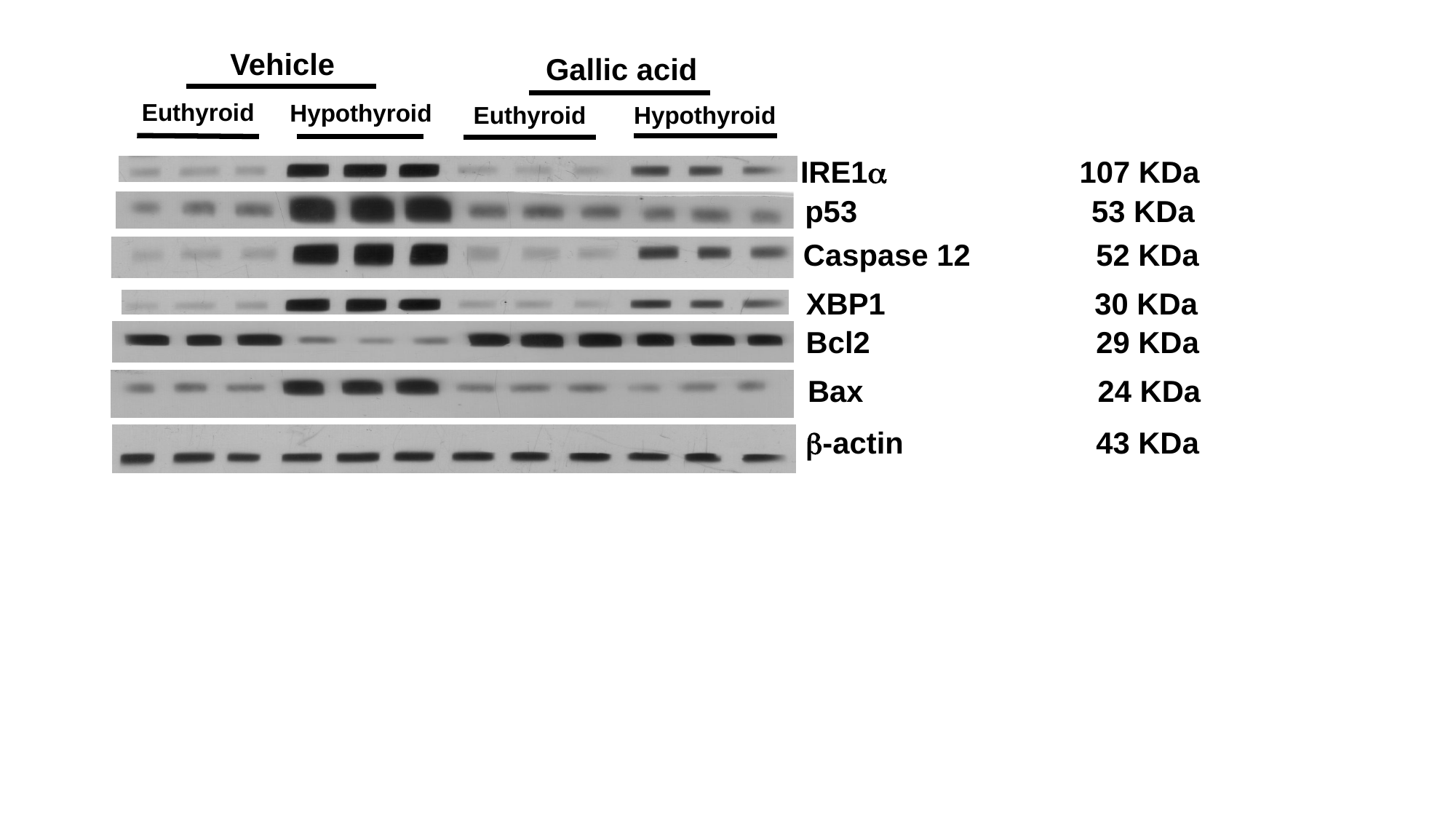

Vehicle
Gallic acid
Euthyroid
Hypothyroid
Euthyroid
Hypothyroid
IRE1a 107 KDa
p53 53 KDa
Caspase 12 52 KDa
XBP1 30 KDa
Bcl2 29 KDa
Bax 24 KDa
b-actin 43 KDa

## Slide 2
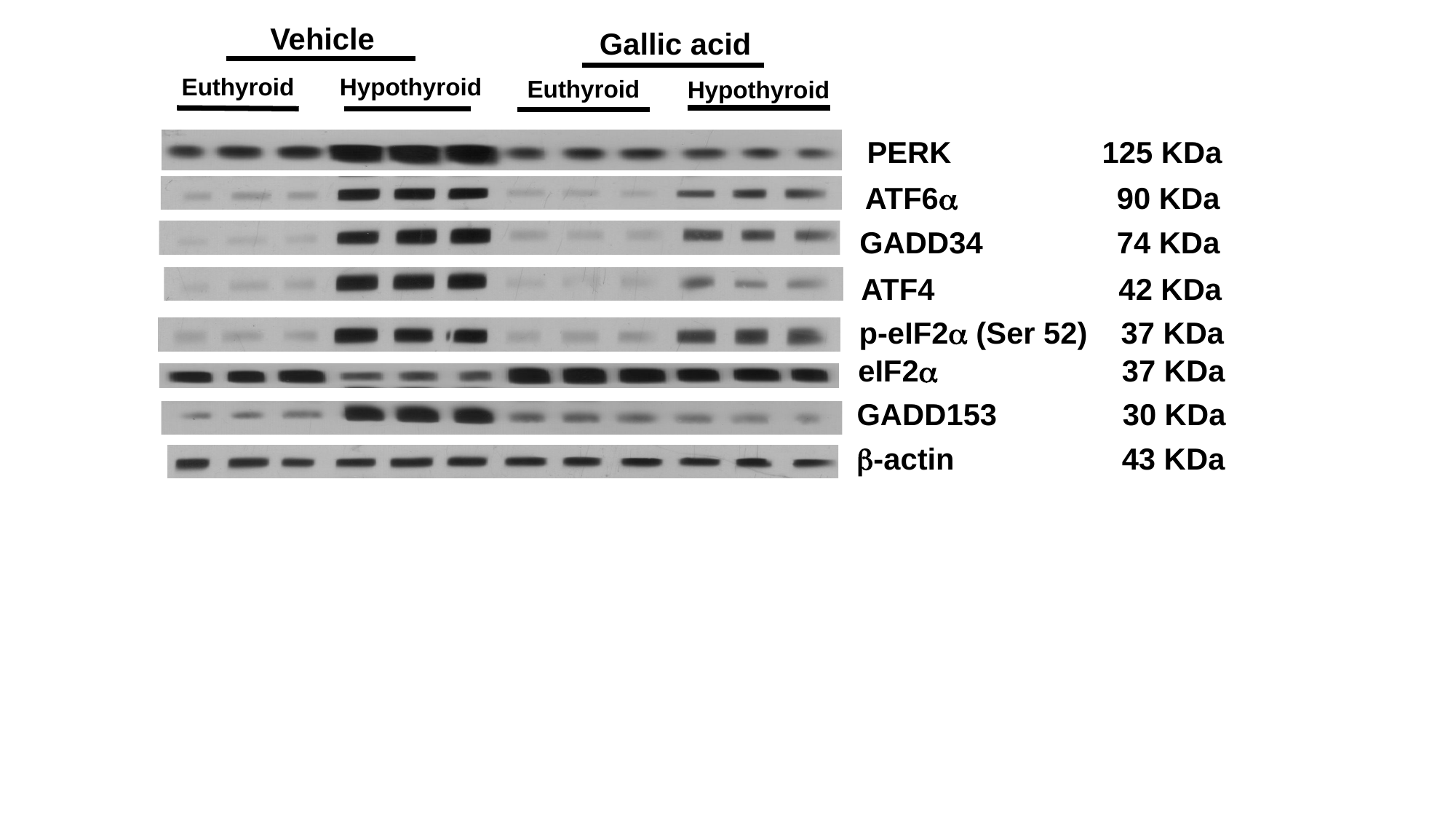

Vehicle
Gallic acid
Euthyroid
Hypothyroid
Euthyroid
Hypothyroid
PERK 125 KDa
ATF6a 90 KDa
GADD34 74 KDa
ATF4 42 KDa
p-eIF2a (Ser 52) 37 KDa
eIF2a 37 KDa
GADD153 30 KDa
b-actin 43 KDa
