# Supplementary figures and images for: Gallic Acid Prevents the Oxidative and Endoplasmic Reticulum Stresses in the Hippocampus of Adult-Onset Hypothyroid Rats
Source: Front Pharmacol. 2021 Jul 6;12:671614. doi: 10.3389/fphar.2021.671614 (PMC8290492; doi:10.3389/fphar.2021.671614)

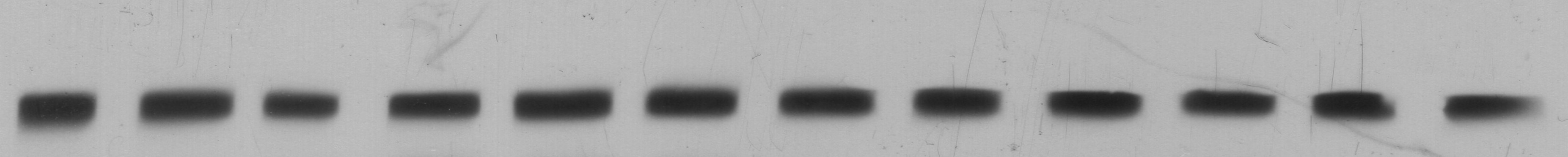

Supplement: Supplementary file 1 [file DataSheet1.ZIP › Actina1.jpg]

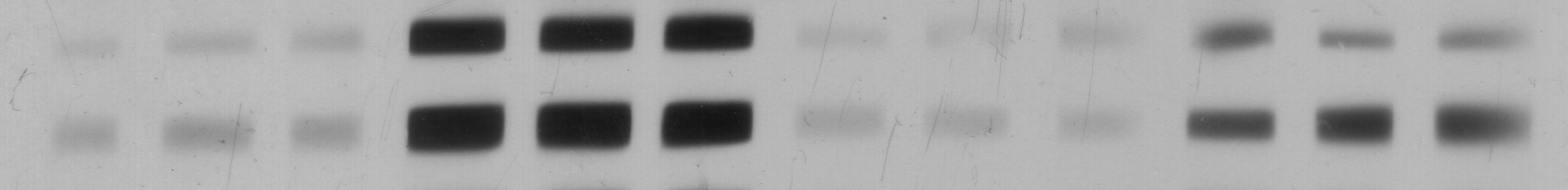

Supplement: Supplementary file 1 [file DataSheet1.ZIP › ATF4-ATF6.jpg]

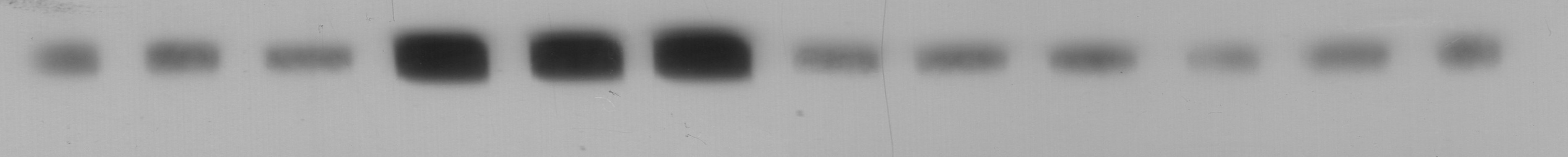

Supplement: Supplementary file 1 [file DataSheet1.ZIP › Bax.jpg]

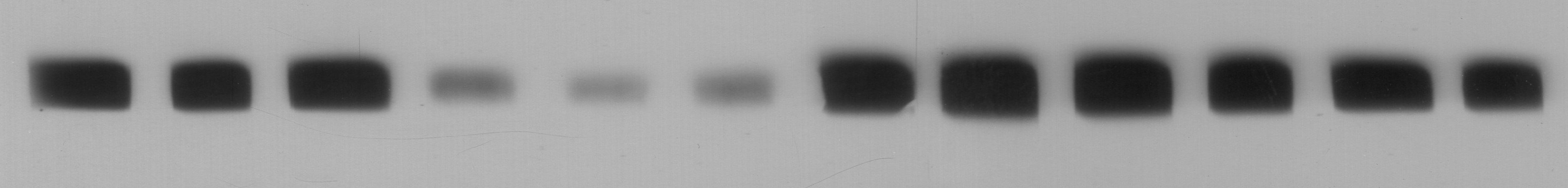

Supplement: Supplementary file 1 [file DataSheet1.ZIP › Bcl2.jpg]

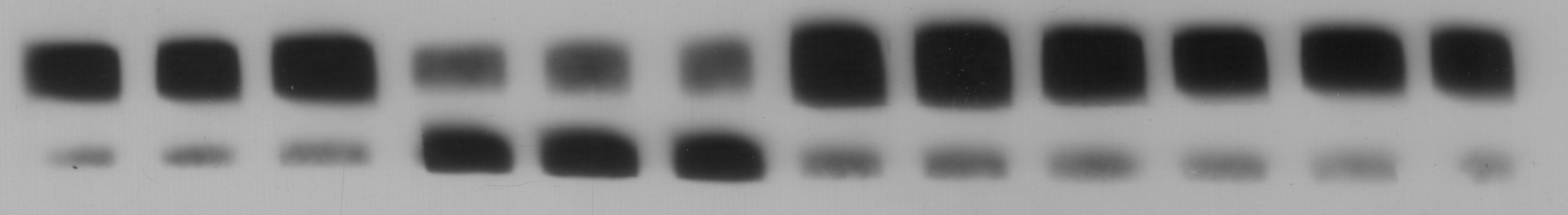

Supplement: Supplementary file 1 [file DataSheet1.ZIP › eIF2a-GADD153.jpg]

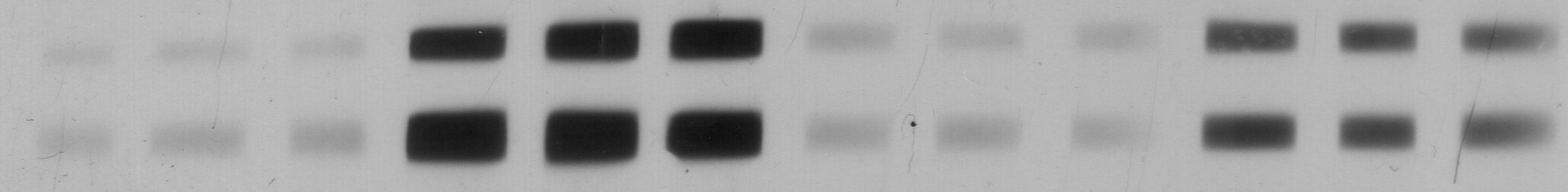

Supplement: Supplementary file 1 [file DataSheet1.ZIP › GADD34-XBP1.jpg]

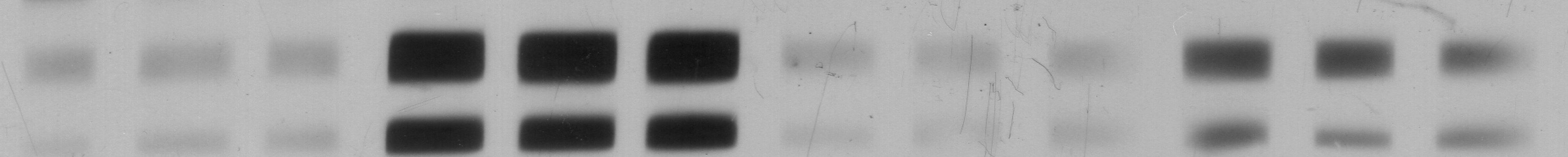

Supplement: Supplementary file 1 [file DataSheet1.ZIP › IRE1a.jpg]

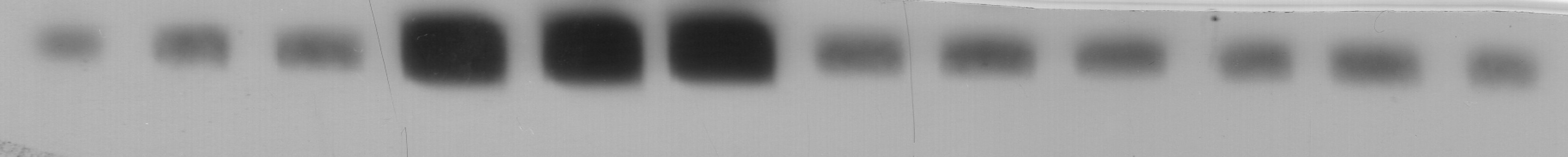

Supplement: Supplementary file 1 [file DataSheet1.ZIP › p53.jpg]

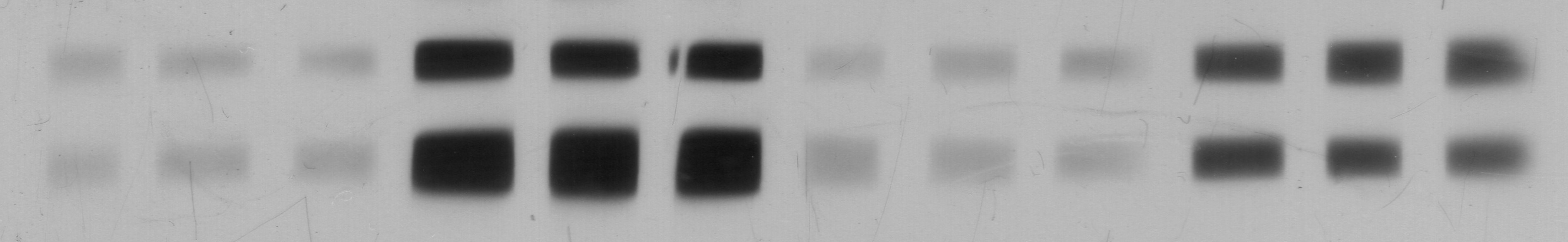

Supplement: Supplementary file 1 [file DataSheet1.ZIP › peIF2a-Caspasa12.jpg]

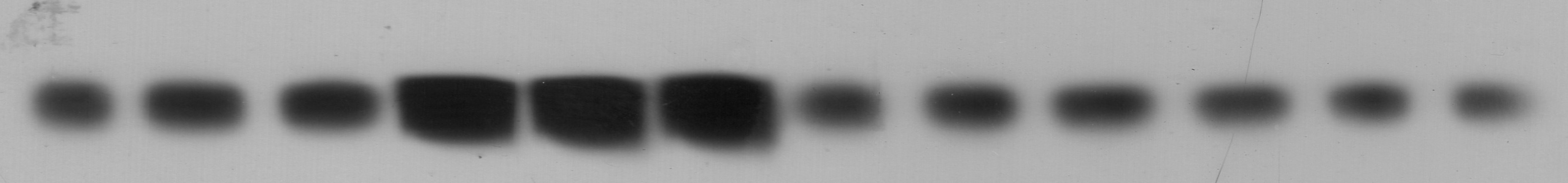

Supplement: Supplementary file 1 [file DataSheet1.ZIP › PERK.jpg]
